# Dynamic Transcriptomic Profiling of Mouse Endometrium Across the Estrous Cycle Reveals Phase‐Specific Regulatory Networks Underlying Cyclic Remodelling

**DOI:** 10.1111/jcmm.71265

**Published:** 2026-06-26

**Authors:** Xinyan Wang, Keyi Xu, Binting Li, Xiaotong Xu, Gang Lu, Le Wang, Ruolang Pan, Ting Zhang

**Affiliations:** ^1^ Department of Gynaecology and Obstetrics The First Affiliated Hospital of Zhejiang Chinese Medical University (Zhejiang Provincial Hospital of Chinese Medicine) Hangzhou China; ^2^ The First Affiliated Hospital of Zhejiang Chinese Medical University (Zhejiang Provincial Hospital of Traditional Chinese Medicine) Hangzhou China; ^3^ Zhejiang Chinese Medical University Hangzhou China; ^4^ Key Laboratory of Cell‐Based Drug and Applied Technology Development in Zhejiang Province Institute for Cell‐Based Drug Development of Zhejiang Province, S‐Evans Biosciences Hangzhou China

**Keywords:** cyclic remodelling, endometrium, hub genes, mouse estrous cycle, transcriptomics

## Abstract

The mouse estrous cycle drives cyclical remodelling of the endometrium, essential for uterine function and embryo implantation. However, the molecular mechanisms orchestrating these dynamic changes remain incompletely defined. In this study, we preliminarily staged C57BL/6 mice using vaginal smear cytology and confirmed phase‐dependent morphological changes, including cyclical endometrial thickening and glandular remodelling, via histological assessment. To elucidate the underlying molecular landscape, we performed comprehensive RNA sequencing of the mouse endometrium across four distinct phases: proestrus, estrus, metestrus and diestrus. To ensure analytical rigour and identify core regulatory factors, we integrated three synergistic computational approaches: WGCNA to identify phase‐specific co‐expression modules, MFUZZ to characterize dynamic temporal expression trajectories, and DESeq2 to pinpoint significantly differentially expressed genes (DEGs). Functional enrichment analysis revealed that these phase‐specific signatures are predominantly involved in the cell cycle, extracellular matrix organization, oxidative phosphorylation and DNA replication. Notably, we identified several high‐connectivity hub genes and pathways, including Natural Killer (NK) cell‐mediated cytotoxicity (*Cd244a*, *Klra4*, *Klrd1*), Oestrogen signalling (*Ccnd1*, *Akt1*, *Prkaca*) and Wnt signalling components (*Cdh1*, *Skp1a*, *Pax2*). Transcription factors such as *Sox4* and *Lef1*, along with the cell cycle regulator *Cks1b*, exhibited phase‐consistent expression patterns aligned with endometrial proliferation and preparation for implantation. Our findings delineate a complex hub‐gene regulatory network coordinating cyclic endometrial regeneration. This study provides a comprehensive transcriptomic atlas of the murine endometrium, offering novel insights into uterine biology with significant implications for understanding reproductive health and uterine diseases.

## 1 Introduction

The mammalian endometrium is a highly dynamic tissue that undergoes cyclical remodelling to prepare for embryo implantation and maintain reproductive competence throughout the reproductive lifespan [1]. This remodelling involves coordinated processes of proliferation, differentiation, tissue breakdown and regeneration, which are tightly regulated by fluctuating levels of ovarian steroid hormones, primarily oestrogen and progesterone [2, 3]. In mice, the estrous cycle lasts approximately 4–5 days and consists of four distinct phases: proestrus, estrus, metestrus and diestrus. Each phase is characterized by unique hormonal milieus and corresponding morphological and molecular changes in the endometrium. During proestrus and estrus, rising oestrogen levels promote endometrial proliferation and thickening, preparing the uterus for potential implantation [4, 5]. Subsequently, during metestrus and diestrus, progesterone predominates, inducing differentiation and functional maturation of the endometrium, followed by regression if fertilization does not occur [6, 7]. Unlike the human menstrual cycle, which involves shedding of the functional endometrial layer, the mouse endometrium does not undergo menstruation but instead exhibits cyclical thickening and thinning through repeated cycles of cell proliferation and apoptosis, reflecting a regenerative process without overt tissue shedding [8, 9].

While morphological and hormonal characterizations of the mouse estrous cycle are well established, the comprehensive molecular landscape underlying cyclic endometrial remodelling remains incompletely defined. Previous transcriptomic studies have revealed that approximately 10% of protein‐coding genes in the mouse uterus are differentially expressed between proestrus and estrus, encompassing pathways involved in ECM remodelling, cell cycle regulation, Wnt and Hedgehog signalling, and immune responses [10, 11]. Many of these pathways show parallels to changes observed during the human menstrual cycle, although temporal alignment differs due to species‐specific reproductive strategies. Single‐cell transcriptomic analyses have further delineated dynamic changes in epithelial, stromal and immune cell populations across the estrous cycle, identifying key transcription factors and signalling networks that govern cellular transitions from regenerative to maturational states [12]. Despite these advances, a full transcriptomic profile spanning all four phases of the mouse estrous cycle, integrating gene expression dynamics with functional pathway analyses, is lacking. Such comprehensive data are essential to elucidate the complex regulatory networks that coordinate cyclical endometrial remodelling and to provide insights into uterine pathophysiology, including infertility, endometriosis and hormone‐dependent cancers.

**FIGURE 1:**
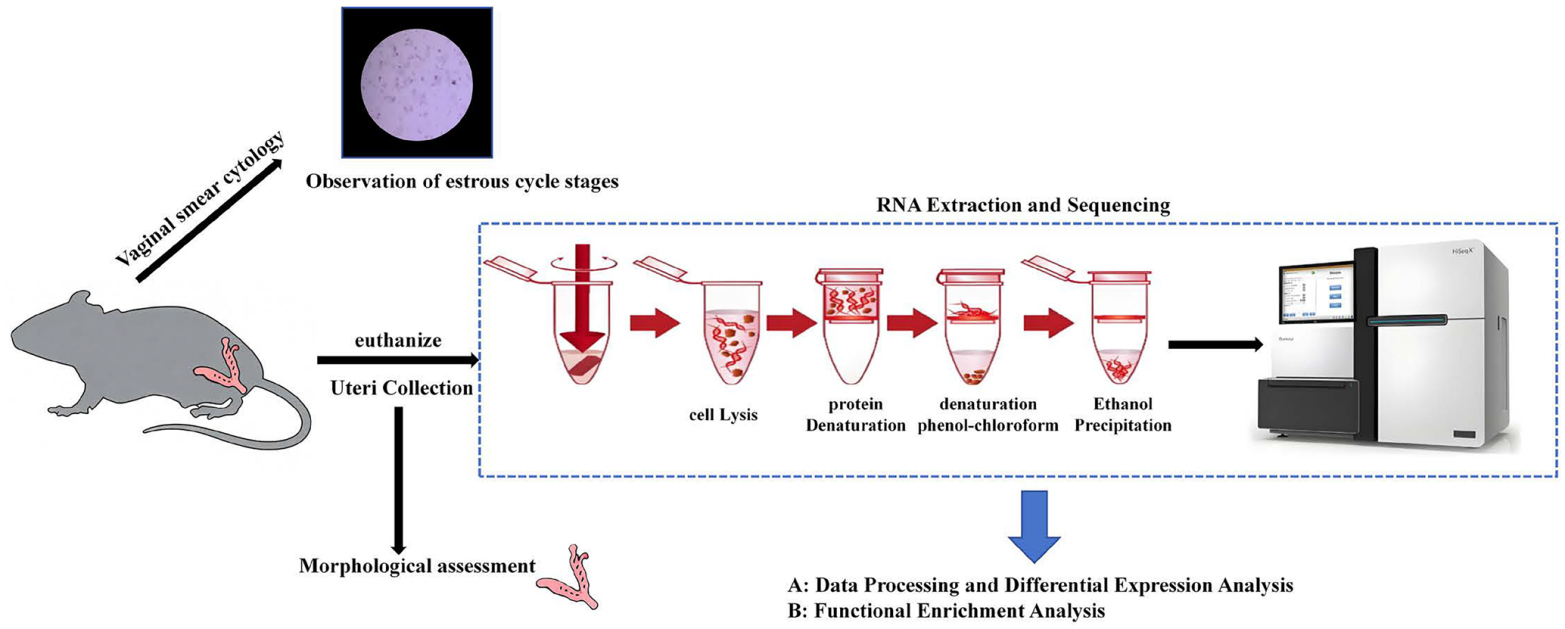
Schematic diagram of workflow for a complex gene regulatory network coordinating cyclic endometrial regeneration. The diagram highlights key steps, including observation of estrous cycle stages, uteri morphological assessment, RNA extraction and subsequent analyses.

In this study, we performed detailed RNA sequencing (RNA‐seq) of mouse endometrium collected at proestrus, estrus, metestrus and diestrus. By combining histological assessment with the hub differentially expressed genes obtained through WGCNA, MFUZZ and DESeq2 methods, we aimed to identify key molecular players and regulatory networks driving cyclic remodelling. Our work provides a valuable resource for understanding the molecular basis of endometrial physiology and establishes a foundation for future research into reproductive health and uterine disease mechanisms. The main workflow of the current study is illustrated in Figure 1.

## 2 Materials and Methods

### 2.1 Animals and Estrous Cycle Staging

Female C57BL/6 mice aged 12–15 weeks were sourced from Vital River Laboratories in Beijing, China, and housed under controlled conditions with ad libitum access to food and water. Estrous cycle stages were determined by daily vaginal smear cytology (9:00 AM). Samples were classified into four phases based on established cellular criteria: (1) Proestrus: predominance of nucleated epithelial cells; (2) Estrus: clusters of cornified anucleated epithelial cells; (3) Metestrus: a mixture of leukocytes, cornified and nucleated epithelial cells; and (4) Diestrus: a predominance of leukocytes. Only mice showing at least two consecutive normal 4–5 day cycles were included in the study. All experimental procedures were performed according to the ‘Guidelines for the Care and Use of Laboratory Animals’ approved by the local Medical Animal Experiment Ethics Committee (ZJCLA, No. ZJCLA‐IACUC‐20050016).

### 2.2 Tissue Collection and Histology

For each estrous stage, a total of 9 mice were used, subdivided into three biological replicates (*n* = 3 per stage). To minimize inter‐individual biological variation and ensure sufficient RNA yield, each biological replicate consisted of pooled endometrial tissues from three individual mice. Consequently, the pool was defined as the statistical unit for all subsequent transcriptomic analyses. Mice were euthanized by an intraperitoneal overdose of pentobarbital (150 mg/kg, Sigma, USA), followed by uterine harvesting. Endometrial tissues were fixed in 4% paraformaldehyde, embedded in paraffin, sectioned (5 μm) and stained with haematoxylin and eosin (H&E) for morphological assessment. Endometrial thickness, gland number and uterine lumen structure were evaluated microscopically. To control for circadian variation in hormone levels and gene expression, all tissue collections were strictly performed between 9:00 AM and 11:00 AM.

### 2.3 RNA Extraction and Library Preparation

Total RNA was extracted from the dissected endometrial tissues of each replicate group using TRIzol reagent (Invitrogen, USA) according to the manufacturer's instructions. RNA integrity and potential DNA contamination were assessed via agarose gel electrophoresis. RNA purity was monitored using a NanoPhotometer spectrophotometer (IMPLEN, USA) based on the OD260/280 and OD260/230 ratios, while RNA integrity was precisely quantified using an Agilent 2100 Bioanalyzer (Agilent Technologies, USA). Poly(A) tail‐containing mRNA was enriched from the total RNA using Oligo(dT) magnetic beads. Subsequently, sequencing libraries were constructed using the NEBNext Ultra RNA Library Prep Kit for Illumina (NEB, USA) following the provider's protocol. The constructed libraries were initially quantified using a Qubit 2.0 Fluorometer (Life Technologies, USA) and diluted to 1.5 ng/μL. The insert size of the libraries was verified using the Agilent 2100 Bioanalyzer. Upon confirming that the insert size met expectations, the effective concentration of the libraries was accurately quantified via qRT‐PCR (ensuring an effective concentration > 2 nM) to guarantee library quality.

### 2.4 RNA‐Seq Data Processing, Alignment and Quantification

Following rigorous quality control, the 12 qualified libraries, corresponding to the four distinct estrous phases with three biological replicates each, were subjected to high‐throughput sequencing on an Illumina platform. Raw image data generated by the sequencer were converted into sequence reads in FASTQ format via CASAVA base calling. The quality of raw sequencing reads was assessed using FastQC (version 0.11.9), focusing on metrics such as Q20, Q30 and GC content (Figure S1). Adapters and low‐quality sequences were trimmed using Cutadapt (version 3.5) to generate clean reads (quality control data pie chart, Figure S2). The resulting clean reads were subsequently aligned to the mouse reference genome (GRCm38, Ensembl release 102) using HISAT2 (version 2.2.1) with default parameters to obtain genomic mapping coordinates. To ensure high‐quality quantification, alignment reads with a mapping quality value below 10, unpaired reads and multi‐mapped reads were filtered out. For gene expression quantification, the number of reads assigned to each gene was subsequently counted using the featureCounts (version 1.5.0‐p3) tool from the Subread package, providing the primary input matrix for differential expression analysis. Concurrently, transcript assembly and abundance normalization were performed using StringTie (version 1.3.3b) to calculate Fragments Per Kilobase Million (FPKM) and Transcripts Per Million (TPM) values, which were used for visualization and downstream WGCNA and MFUZZ clustering analysis.

### 2.5 Bioinformatics and Differential Expression Analysis

Differential expression analysis was performed using the DESeq2 R package (version 1.34.0). After filtering out genes with zero counts across all samples, a total of 33,599 genes were retained for downstream analysis. Significantly differentially expressed genes (DEGs) were identified between adjacent estrous phases (E vs. PRO, MET vs. E, DI vs. MET and DI vs. PRO) based on the strict criteria of |log_2_FC| ≥ 1 and adjusted *p*‐value < 0.05. To characterize global expression trajectories, temporal clustering was performed using the MFUZZ R package (version 2.72.0), assigning genes into 10 distinct clusters based on their expression patterns across the four phases. Furthermore, a weighted gene co‐expression network was constructed using the WGCNA R package (version 1.74.0). After filtering low‐expression genes, the Topological Overlap Matrix (TOM) was calculated, resulting in the identification of 13 co‐expression modules. The six modules most significantly correlated with specific estrous stages were selected for hub gene identification and functional pathway enrichment analysis.

### 2.6 Functional Enrichment Analysis

Gene Ontology (GO) and Kyoto Encyclopedia of Genes and Genomes (KEGG) [13, 14] pathway enrichment analyses were performed on DEGs to identify overrepresented biological processes, cellular components, molecular functions and signalling pathways (adj *p* < 0.05).

### 2.7 qRT‐PCR Validation

Candidate genes representing key pathways were selected for validation. cDNA was synthesized from RNA samples, and quantitative real‐time PCR was performed using gene‐specific primers. Relative expression levels were calculated using the 2^−ΔΔ*Ct*^ method, normalized to housekeeping genes.

## 3 Results

### 3.1 Phenotypic Characteristics of Mouse Endometrium at Different Estrous Cycle Stages

**FIGURE 2:**
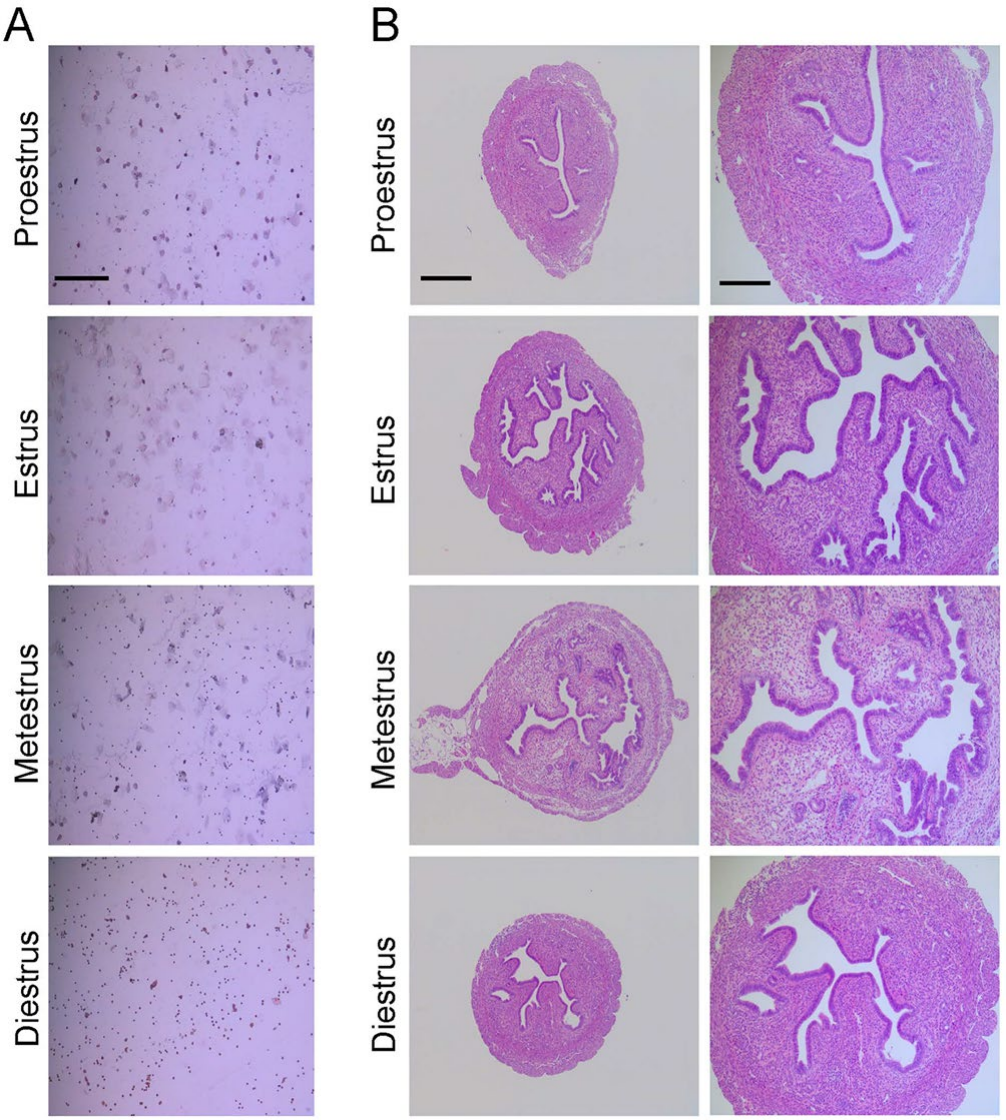
Cytological and histological characterization of endometrial morphology during the four estrous phases. (A) Estrous cycle stages were determined by vaginal smear cytology. Scale bar = 100 μm. (B) Histological examination of uterine tissue sections stained with haematoxylin and eosin. Left panels show low magnification (scale bar = 200 μm), and right panels show higher magnification focusing on glandular structures (scale bar = 100 μm).

To preliminarily determine the estrous cycle stages of C57BL/6 mice, vaginal smear cytology was performed. As shown in Figure 2A, typical cytological features were observed for each stage: Proestrus was characterized by a predominance of nucleated epithelial cells and few anucleated keratinized cells. Estrus showed abundant anucleated keratinized cells with fewer nucleated epithelial cells. Metestrus presented a mixture of nucleated epithelial cells, anucleated keratinized cells and leukocytes. Diestrus was dominated by leukocytes with only occasional nucleated epithelial cells. These cytological patterns align well with established criteria and reflect the hormonal fluctuations underlying the estrous cycle. Histological examination of uterine tissue sections stained with haematoxylin and eosin further confirmed cyclical morphological changes in the endometrium (Figure 2B). Compared to diestrus, the endometrium was thinner and the uterine lumen simpler during proestrus, with a smaller endometrial area. During estrus, the endometrium became swollen, the uterine lumen enlarged and more complex, and the number of endometrial glands increased significantly. In metestrus, although the uterine lumen maintained a complex structure and the endometrial area remained large, the number of glands began to decrease, and no further expansion of the lumen was observed. By diestrus, the endometrium thinned again, the uterine lumen simplified, the endometrial area decreased, and gland numbers rapidly declined. This cyclical thickening and thinning of the mouse endometrium parallels the human menstrual cycle in terms of tissue remodelling, although mice do not undergo menstrual shedding but rather repeated cycles of proliferation and apoptosis [15]. The endometrial structure during metestrus closely resembled that of estrus, with sufficient thickness and glandular density to support embryo implantation. This period corresponds to the mouse implantation window, a critical phase for reproductive success [16].

**FIGURE 3:**
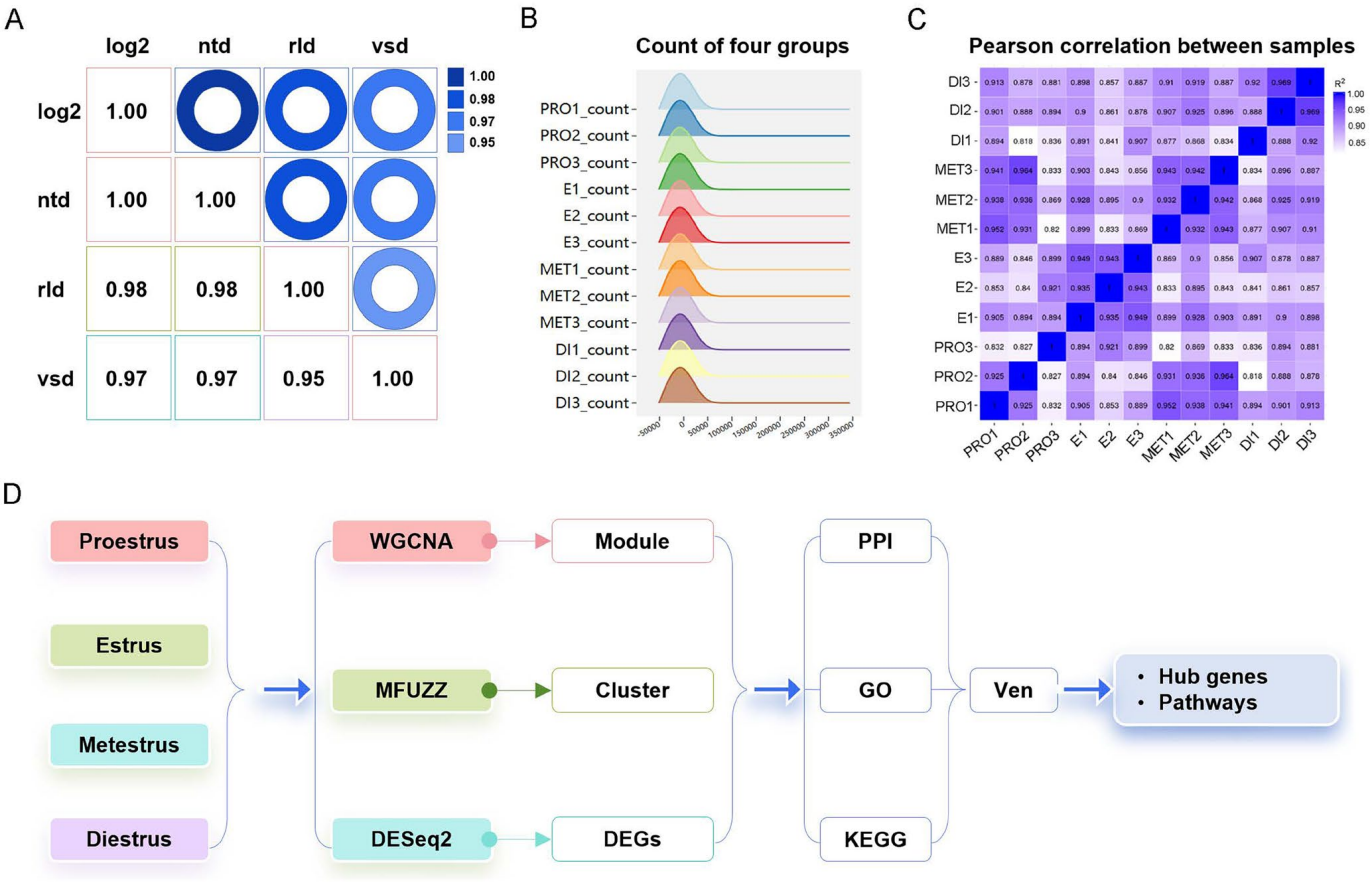
Data reliability and biological replicates analysis, and data analysis workflow. (A) Differences in correlation coefficients of statistical expression matrices normalized by different methods under four algorithms of the dataset. The correlation of all four algorithms reached above 0.94. (B) Ridge plot showing the count values of 33,599 genes in 12 samples. (C) Pearson correlation between samples. (D) Schematic diagram of transcriptome data analysis workflow (including module screening under WGCNA; cluster screening under MFUZZ time‐series analysis; DEG screening under DESeq2 algorithm) (log_2_FC ≥ 1 or log_2_FC ≤ −1, adj *p* < 0.05).

Following staging via vaginal cytology, endometrial tissues were collected from mice at each estrous phase for transcriptomic profiling. To ensure robust sampling, three biological replicates were established for each of the four phases, with each replicate consisting of pooled tissues from three individual mice. This resulted in 12 sequenced samples: proestrus (PRO1, PRO2, PRO3), estrus (E1, E2, E3), metestrus (MET1, MET2, MET3) and diestrus (DI1, DI2, DI3). To ensure data reliability, raw counts were normalized using four distinct algorithms, all of which yielded high inter‐method correlations (> 95%) (Figure 3A). Furthermore, a ridge plot of the 33,599 detected genes (Figure 3B) and a Pearson correlation heatmap confirmed strong intra‐group consistency among biological replicates. Pearson correlation analysis of sample pairs, quantified by the squared correlation coefficient (*R* ^2^), revealed strong correlations (*R* ^2^ > 0.88) among replicates within each group, except for PRO3, which had lower correlations with PRO1 and PRO2 (*R* ^2^ = 0.832 and 0.827, respectively) (Figure 3C). This suggests high reproducibility of sampling and sequencing, with minor variability possibly reflecting transitional transcriptional states during proestrus.

Collectively, these findings validate the accuracy of vaginal cytology for estrous staging and characterize the distinct morphological and molecular landscapes of the murine endometrium across the estrous cycle. The observed cyclical changes in endometrial thickness, gland number and tissue architecture correspond to known hormonal fluctuations and prepare the uterus for potential embryo implantation during the metestrus phase [17, 18]. This phenotypic characterization establishes a robust foundation for systematic molecular investigations into endometrial remodelling. To identify key hub genes and their expression dynamics across the cycle, we implemented an integrated bioinformatic framework comprising WGCNA, MFUZZ and DESeq2 analyses (Figure 3D).

### 3.2 Identification of Stage‐Specific Co‐Expression Modules via WGCNA

**FIGURE 4:**
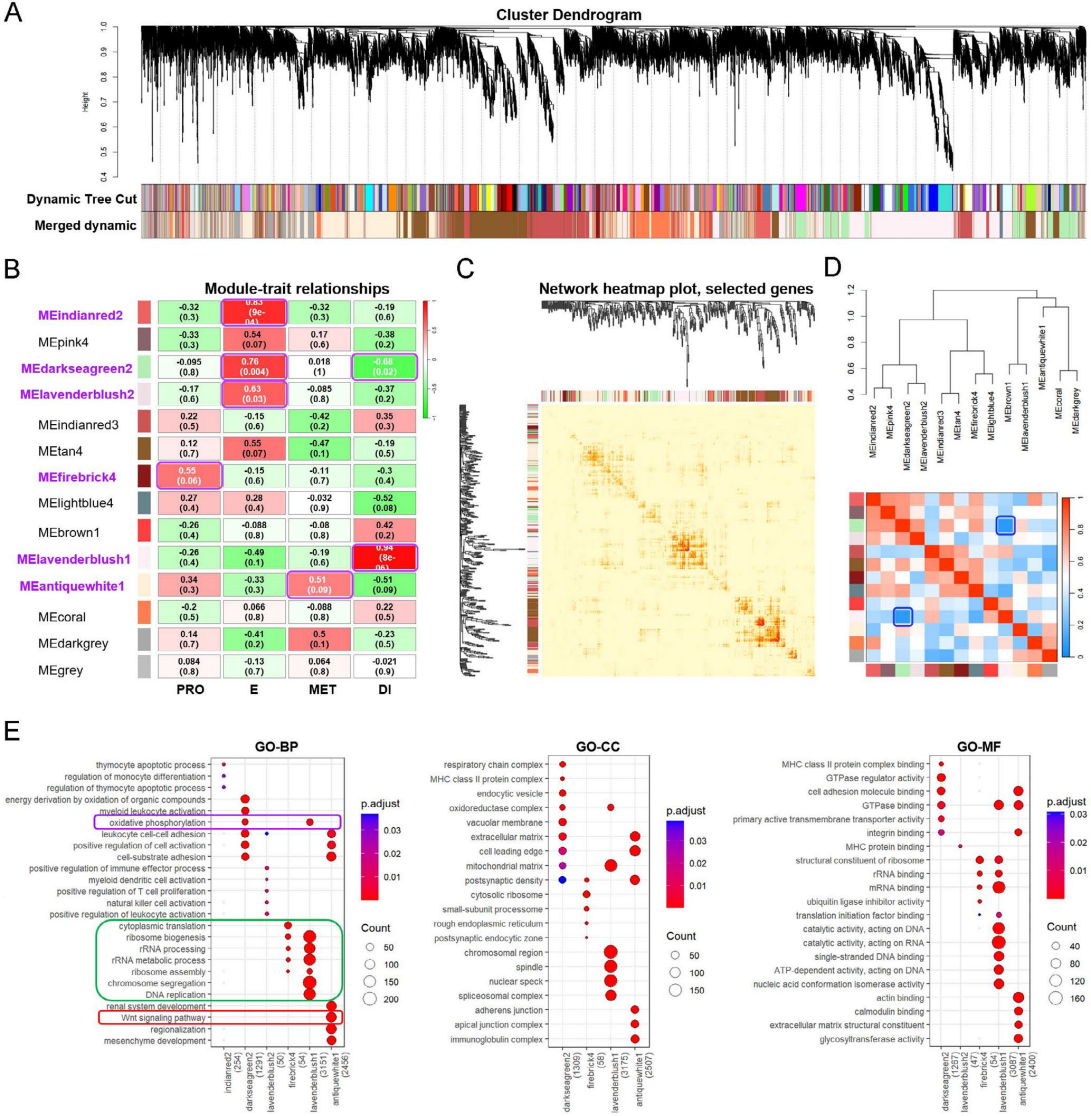
Results of weighted gene co‐expression network analysis and subsequent gene ontology (GO) enrichment analysis. (A) Cluster dendrogram. This chart can be viewed in two parts: The upper part is the hierarchical clustering dendrogram of genes, and the lower part is the gene modules, that is, the network modules. The upper and lower parts correspond, showing that genes that are closer in distance (clustered on the same branch) are assigned to the same module. ‘Merged’ is the colour after similar modules are combined. (B) Module‐trait associations. Each row corresponds to a module eigengene, column to a trait. Each colour contains the corresponding correlation and *p*‐value. The purple modules are the selected modules. (C) Visualizing the gene network using a heatmap plot. Heatmap depicts the Topological Overlap Matrix (TOM) among all genes in the analysis. (D) Visualization of the eigengene network representing the relationships among the modules and the four trait weight. Darkseagreen2 and lavenderblush1 modules are negatively correlated. (E) GO enrichment analysis results of selected multiple modules.

To elucidate the systemic gene association patterns governing endometrial remodelling, we employed Weighted Gene Co‐Expression Network Analysis (WGCNA). This systems biology approach identifies highly co‐varying gene sets (modules) and prioritizes candidate biomarkers based on their intramodular connectivity and phenotypic associations. Based on a scale‐free topology analysis of the 12 samples, a soft‐thresholding power of 19 was selected (Figure S3), resulting in the identification of 13 distinct co‐expression modules (Figure 4A–D). Notably, the darkseagreen2 module exhibited highly divergent correlations between the estrus (E) and diestrus (DI) phases, suggesting a functional reversal of the underlying biological processes during these two stages. While correlation heatmap analysis revealed a strong negative relationship between the brown1 and darkseagreen2 modules, the brown1 module itself did not show significant correlations with any specific estrous stage. Subsequent Gene Ontology (GO) enrichment analysis of the modules most highly correlated with specific phases provided further functional insights (Figure 4E). The indianred2 module was primarily enriched in the thymocyte apoptotic process and monocyte differentiation. The darkseagreen2 module was involved in oxidative phosphorylation, cell activation and respiratory chain complexes. Meanwhile, the antiquewhite1 module showed significant enrichment in the Wnt signalling pathway, regionalization and extracellular matrix (ECM) structural constituents.

**FIGURE 5:**
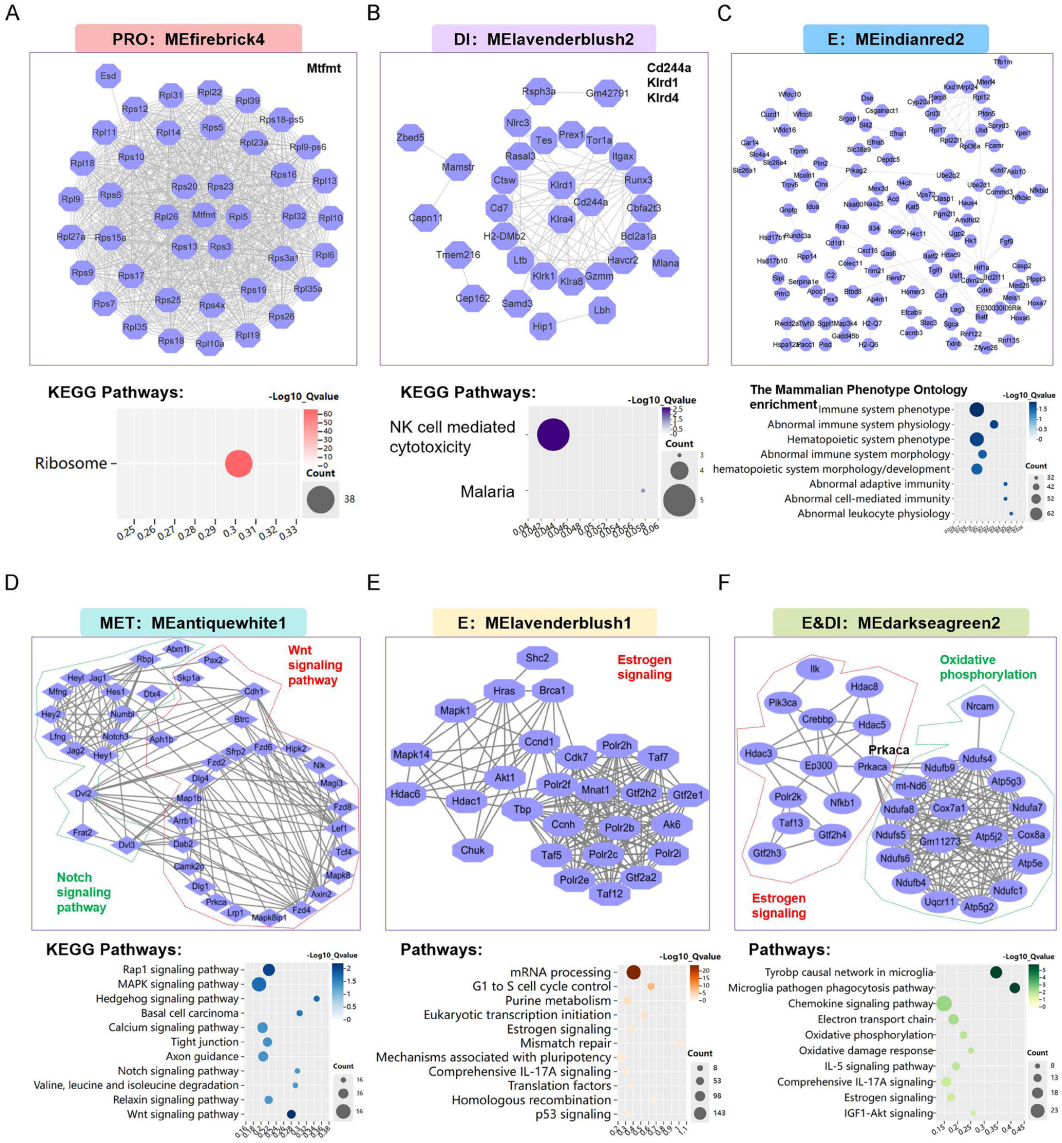
Interaction networks for the six primary colour‐coded modules were constructed to resolve the regulatory architecture. (A) PRO‐related firebrick4 module interaction network gene map and the regulated signalling pathway display in the enrichment analysis results (Ribosome). (B) DI‐related lavenderblush2 module interaction network gene map and the regulated signalling pathway display in the enrichment analysis results (NK cell mediated cytotoxicity). (C) E‐indianred2 module interaction network gene map and the regulated signalling pathway display in the enrichment analysis results (Immune system). (D) MET‐related antiquewhite1 module interaction network gene map and the regulated signalling pathway display in the enrichment analysis results (Notch signalling pathway, Want signalling pathway). (E) E‐related lavenderblush1 module interaction network gene map and the regulated signalling pathway display in the enrichment analysis results (Oestrogen signalling). (F) E&DI‐related darkseagreen2 module interaction network gene map and the regulated signalling pathway display in the enrichment analysis results (Oestrogen signalling, Oxidative phosphorylation).

To further resolve the regulatory architecture, we constructed interaction networks for the six primary colour‐coded modules (Tables S1 and S2, Figure 5A–F). In the proestrus (PRO)‐related firebrick4 module, we identified *Mtfmt* (Mitochondrial methionyl‐tRNA formyltransferase) as a pivotal gene interacting within the ribosome pathway, suggesting a heightened state of mitochondrial protein synthesis initiation during this phase. Other phase‐specific interactions included the diestrus (DI)‐related lavenderblush2 module, which was associated with Natural Killer (NK) cell‐mediated cytotoxicity, and the estrus (E)‐related indianred2 module, which correlated with immune system regulation. The metestrus (MET)‐related antiquewhite1 module featured critical interactions within the Notch and Wnt signalling pathways, including the key transcription factor *Lef1*, a well‐known regulator of organ development and uterine glandular function.

Notably, Oestrogen signalling and oxidative phosphorylation pathways were associated with both the lavenderblush1 and darkseagreen2 modules; however, their correlation directions were diametrically opposed between the E and DI phases (Figure 5F). This indicates that Oestrogen signalling is highly active during estrus but enters a dormant state during diestrus, a pattern that aligns closely with the physiological fluctuations of the estrous cycle. We identify the darkseagreen2 module as the most biologically significant, as it appears to coordinate the metabolic and developmental trends essential for endometrial homeostasis. A central hub within this landscape is *Prkaca* (Protein Kinase cAMP‐Activated Catalytic Subunit Alpha). Given that *Prkaca* regulates a wide array of functional proteins, including metabolic enzymes, transcription factors and cell cycle regulators in a signal‐dependent manner, it likely serves as a critical node in orchestrating cyclic endometrial regeneration.

### 3.3 Differential Gene Expression and Functional Landscape of the Endometrium Across the Estrous Cycle

**FIGURE 6:**
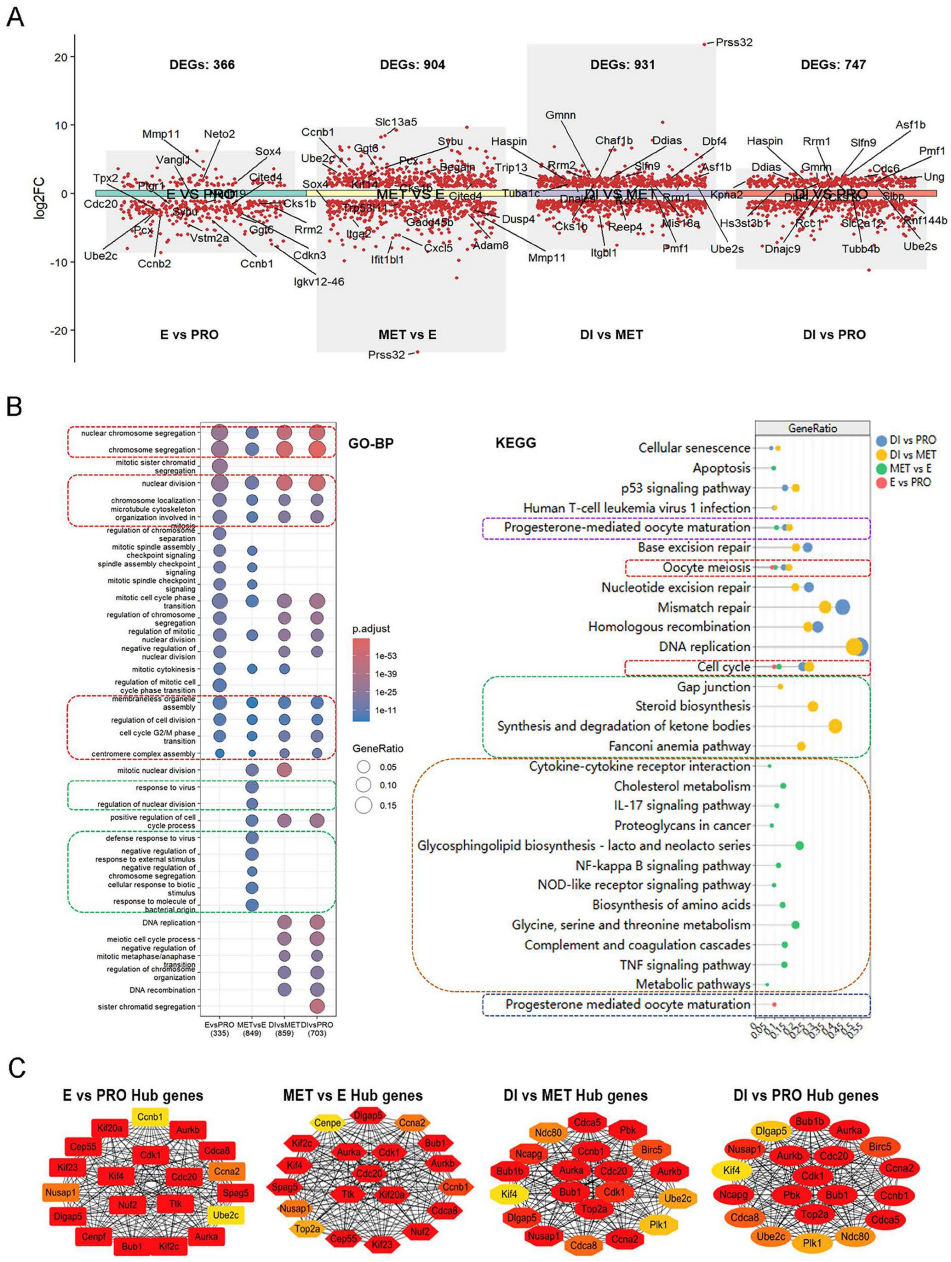
Analysis of differential gene expression and functional landscape of the endometrium across the estrous cycle. (A) Volcano plot of differentially expressed genes (DEGs) between groups (log_2_FC ≥ 1 or log_2_FC ≤ −1, adj *p* < 0.05). (B) GO‐BP and KEGG multi‐group enrichment analysis results of inter‐group differential genes. (C) The top 20 genes in the intergroup gene interaction network (PPI interaction network plugin Hub Gene plotting).

Prior correlation and principal component analysis (PCA) confirmed strong reproducibility among biological replicates within each group, with the minor exception of sample PRO3, which exhibited a slight deviation while remaining within the proestrus cluster. Notably, the transcriptomic profiles of proestrus and metestrus displayed significant convergence, suggesting that genes upregulated during the transition from proestrus to estrus tend to revert toward baseline levels by the metestrus phase, thereby reflecting the cyclical nature of endometrial remodelling. To identify phase‐specific signatures, we performed pairwise differential expression analysis between adjacent estrous stages using DESeq2 (FPKM, Table S3; the TPM values were provided for peer reference, Table S4). Based on stringent criteria (adj *p* < 0.05 and |log_2_ fold change| ≥ 1), we identified a total of 2087 unique DEGs (Table S5). The transitions involved varying degrees of transcriptional shifts: 366 DEGs were identified in E vs. PRO, 904 in MET vs. E, 931 in DI vs. MET and 747 in DI vs. PRO (Figure 6A). To validate the RNA sequencing results, 12 genes (*Wnt5α*, *Wnt7b*, *Hif1α*, *Hnf1α*, *Cdk1*, *Krt8*, *Hoxa10*, *Fgf9*, *Nid1*, *Nog*, *Nrk*, *Pdgfd*) exhibiting sustained differential expression throughout the estrous cycle were selected for quantitative real‐time PCR (qRT‐PCR, Figure S4).

To elucidate the biological implications of these dynamic gene sets, we performed GO (Figure 6B, Figure S5) and Kyoto Encyclopedia of Genes and Genomes (KEGG, Table S6) enrichment analyses. GO biological process (BP) terms enriched across all comparisons exhibited considerable overlap, highlighting shared themes in cyclic remodelling such as chromosome segregation, nuclear division, membraneless organelle assembly and cytoskeleton organization (Figure 6B). Furthermore, KEGG analysis revealed consistent enrichment in pathways essential for tissue turnover, including the cell cycle, DNA replication and oocyte meiosis. Intriguingly, pathways related to progesterone‐mediated oocyte maturation were significantly enriched in the first three transitional stages, underscoring the endometrium's functional response to fluctuating steroid hormones. This active remodelling was further supported by the enrichment of DNA repair mechanisms (base excision and mismatch repair), apoptosis, and immune‐regulatory pathways such as NF‐κB signalling and cytokine‐cytokine receptor interactions.

To identify the central nodes driving these cyclical transitions, we constructed Protein–Protein Interaction (PPI) networks for each comparison using the STRING database (Figure 6C). Hub gene screening revealed a highly consistent set of core regulators across multiple transitions. For instance, the E vs. PRO network was centred on critical cell cycle regulators, including *Cdk1*, *Cdc20*, *Ttk*, *Aurkb* and members of the kinesin family (e.g., *Kif4*, *Kif23*). A similar constellation of hub genes, including *Cdk1*, *Cdc20* and *Bub1*, dominated the MET vs. E transition. The high degree of overlap among these hub genes across different stages suggests that a conserved molecular ‘engine’, characterized by the cyclical oscillation of factors like *Cdk1*, is the primary driver of endometrial proliferation, apoptosis and structural regeneration throughout the estrous cycle.

### 3.4 MFUZZ Temporal Profiling and Hub Gene Identification via Pseudo‐Time Series Analysis

**FIGURE 7:**
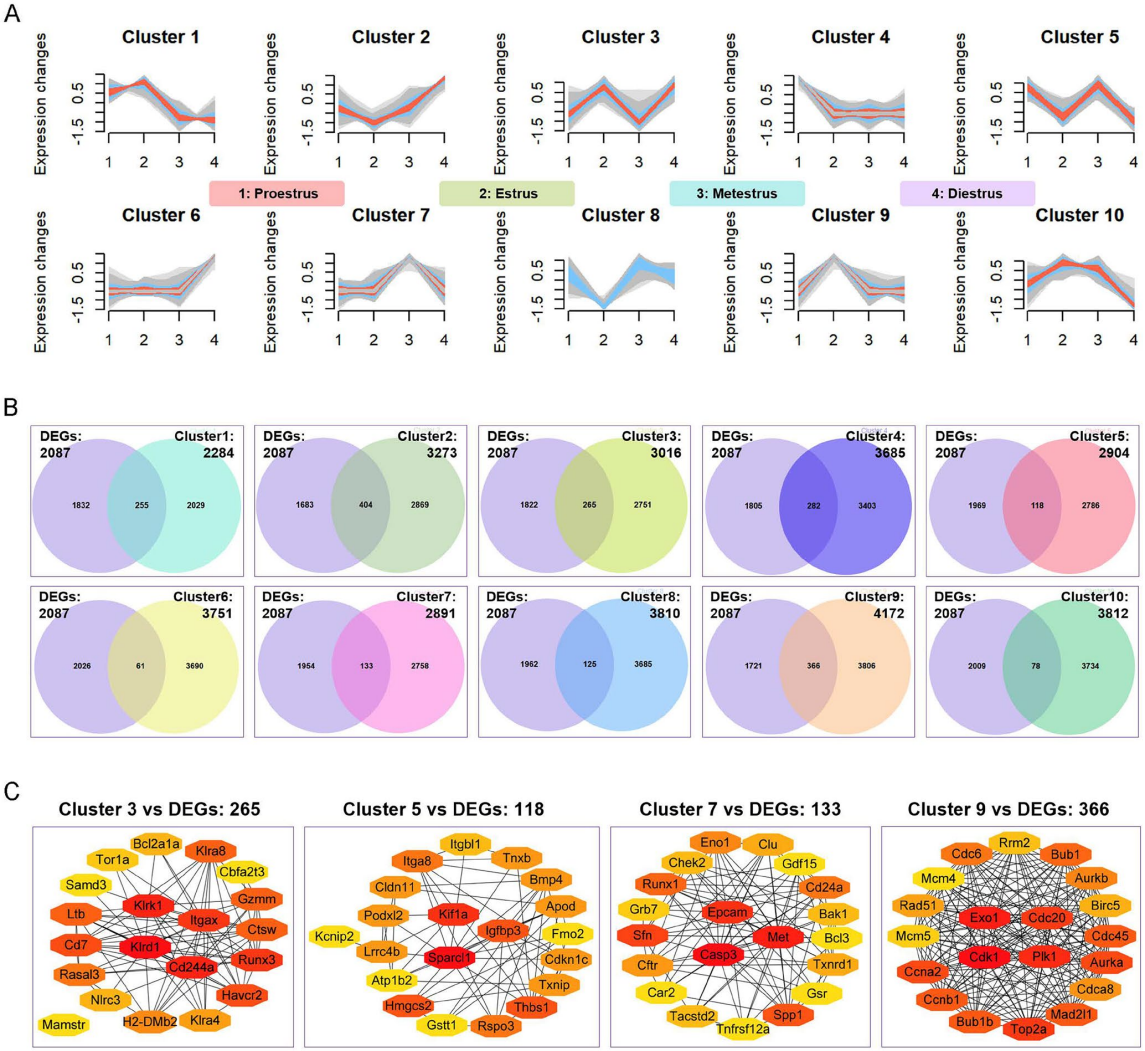
MFUZZ temporal profiling and hub gene identification via pseudo‐time series analysis. (A) MFUZZ results of 33,599 total genes between groups (Define 10 clusters). (B) Take the intersection of genes in 10 clusters and DEGs. (C) The top 20 genes in the intergroup gene interaction network (PPI interaction network plugin Hub Gene plotting). The hub genes selected by MFUZZ, the hub genes of WGCNA modules, and the hub genes among inter‐group differential genes are all highly overlapping and consistent.

To further elucidate the dynamic transcriptional trajectories across the estrous cycle, we performed a soft‐clustering analysis using the MFUZZ R package on the 33,599 quantified genes (Table S7). This temporal profiling identified 10 distinct clusters, each representing a unique expression pattern that characterizes the cyclical nature of the murine endometrium. These trajectories encompass gene sets that exhibit phase‐specific peaks as well as those that remain relatively stable throughout the transition between cycles (Figure 7A).

To prioritize genes with the highest biological relevance, we intersected the gene sets within these 10 clusters with the 2087 DEGs previously identified via DESeq2 (Figure 7B, Tables S8 and S9). From this refined dataset, we focused on Clusters 3, 5, 7 and 9—which exhibited the most dynamic fluctuations—to construct protein–protein interaction (PPI) networks and identify the top 20 hub genes within each cluster (Figure 7C). Notably, these temporal hubs showed substantial overlap with the key regulators identified in our earlier DESeq2 and WGCNA analyses, including *Cdc20*, *Cdk1*, *Igfbp3*, *Klrd1*, *Klrk1* and *Plk1*. This high degree of convergence across multiple bioinformatic pipelines reinforces the functional significance of these core factors in coordinating the periodic proliferation and remodelling of the mouse endometrium.

## 4 Discussion

The cyclical remodelling of the mouse endometrium is a highly coordinated biological event involving dynamic changes in gene expression, cellular proliferation, differentiation and immune modulation. In this study, we provide a comprehensive four‐phase transcriptomic atlas of the murine endometrium. By integrating DESeq2, WGCNA and MFUZZ analyses, we moved beyond simple pairwise comparisons to delineate a system‐level hub‐gene regulatory network that orchestrates these transitions.

Consistent with earlier reports, approximately 10% of the uterine protein‐coding genome exhibits differential expression across the estrous cycle [19], reflecting extensive tissue remodelling. This remodelling involves extracellular matrix (ECM) reorganization, cell cycle activation and modulation of adhesion molecules and immune‐related genes. Our use of pooled biological replicates (*n* = 3 pools, each from 3 mice) was a deliberate strategy to reduce inter‐individual noise and ensure sufficient RNA yield for high‐depth sequencing. However, we explicitly acknowledge that this pooling design may mask individual‐level biological variability and limit the interpretation of variability at the individual animal level. While the PRO3 sample exhibited a slight deviation in PCA, its successful clustering within the proestrus group across multiple algorithms (WGCNA/MFUZZ) suggests it represents a biologically meaningful transitional state rather than a technical outlier. This reflects the inherent fluidity of transcriptional boundaries in a rapidly remodelling tissue. While direct serum hormone measurements were not performed, the high concordance between our histological findings and established cyclicity markers provides strong evidence for the accuracy of our staging. We acknowledge that the absence of biochemical validation is a limitation, and the slight deviation of the PRO3 sample likely reflects the inherent transcriptional fluidity during the rapid transition from diestrus to proestrus.

While the involvement of Wnt signalling in endometrial proliferation is well‐established [20, 21, 22], our integrated transcriptomic profiling provides a more detailed, full‐cycle perspective that goes beyond previously reported two‐phase dynamics. Specifically, our WGCNA analysis identified the antiquewhite1 module as a stage‐specific regulatory unit, with *Lef1* and *Pax2* acting as central hubs. While *Lef1* is a known mediator of uterine gland development, its identification as a high‐connectivity node in our network, coupled with the peak expression of *Cdh1* (E‐cadherin), suggests a more complex role for Wnt signalling in coordinating cell‐to‐cell adhesion and structural integrity across the four phases. Furthermore, the biphasic oscillation of Wnt ligands observed in our study suggests that this pathway does not merely drive estrus‐associated expansion but is also precisely downregulated during diestrus to facilitate functional maturation [23, 24], a temporal precision that has not been fully articulated in earlier rodent models.

A striking discovery was the functional reversal of the darkseagreen2 module between the estrus and diestrus phases. This module, enriched in oxidative phosphorylation and oestrogen signalling, was highly active during estrus but became dormant during diestrus. This metabolic switch is anchored by the hub gene *Prkaca*, a signal‐dependent catalytic subunit that likely serves as a master regulator of the cellular energy shifts required for cyclical regeneration. Concurrent with this metabolic dormancy, Hypoxia‐inducible factor 1‐alpha (HIF‐1α) expression peaked during diestrus [25]. This aligns with its role in mediating responses to low oxygen tension, promoting angiogenesis and stromal survival critical for the transition into the next cycle. HIF‐1α promotes angiogenesis and stromal cell survival, critical for endometrial repair and preparation for subsequent cycles [26, 27, 28]. The interplay between hypoxia signalling and hormonal regulation likely coordinates tissue regeneration and vascular remodelling, as progesterone dominance during diestrus modulates hypoxia‐responsive pathways. Moreover, the crosstalk between HIF‐1α and Wnt signalling pathways may synergistically regulate endometrial cell fate decisions and extracellular matrix remodelling, processes essential for maintaining uterine receptivity and preventing pathological conditions such as endometriosis.

Endometrial remodelling is intrinsically linked to immune system modulation, particularly during the estrus‐to‐metestrus transition, which corresponds to the implantation window [29, 30, 31]. Our data highlighted the recruitment of uterine natural killer (uNK) cells and macrophages via the TNF signalling and leukocyte migration pathways. Crucially, we observed that the balanced expression of cytokines, including Leukaemia Inhibitory Factor (LIF), is essential for a receptive environment [32]. Reduced LIF levels are a hallmark of implantation failure in both rodents and humans [32, 33, 34]. These immune adaptations underscore the delicate balance between pro‐inflammatory and anti‐inflammatory signals required for successful implantation and highlight potential immunological causes of reproductive failure.

The intricate interplay between oestrogen and progesterone drives these molecular events. Oestrogen‐driven proliferation is reflected in the peak expression of cell cycle hubs like *Cdk1* and the growth factor regulator *Igfbp3* [35, 36]. Conversely, the transition to progesterone dominance suppresses Wnt signalling during diestrus, preventing pathological hyper‐proliferation [37, 38]. The integration of our transcriptomic data with histological observations confirms that while the mouse does not undergo menstrual shedding, its regenerative pathways (cell proliferation vs. apoptosis) are molecularly analogous to the human menstrual cycle, while progesterone induces differentiation and decidualization via factors including Hand2 and Bmp2.

In summary, our study delineates a complex, hub‐gene centred regulatory network involving Wnt signalling, hypoxia response and immune modulation that coordinates cyclical endometrial remodelling. By identifying specific nodes like *Prkaca*, *Lef1* and *Cdk1*, we provide a foundation for future mechanistic studies into uterine pathophysiology, including infertility, endometriosis and hormone‐dependent cancers. Understanding how these pathways interact to maintain endometrial homeostasis and receptivity will be critical for improving fertility treatments and managing endometrial disorders.

## Author Contributions

**Xinyan Wang:** investigation, writing – original draft. **Ting Zhang:** conceptualization, writing – review and editing, project administration, supervision, funding acquisition. **Binting Li:** data curation, methodology. **Ruolang Pan:** conceptualization, writing – review and editing, project administration, supervision. **Keyi Xu:** investigation, writing – original draft. **Xiaotong Xu:** data curation, methodology. **Gang Lu:** data curation, validation. **Le Wang:** methodology, investigation.

## Funding

This work was supported by the Joint Funds of the Zhejiang Provincial Natural Science Foundation of China under grant no. LBY24H040008.

## Ethics Statement

All experiments were conducted according to the protocols approved by the local Medical Animal Experiment Ethics Committee (ZJCLA, No. ZJCLA‐IACUC‐20050016).

## Conflicts of Interest

The authors declare no conflicts of interest.

## Supporting information


**Table S1:** Lists of WGCNA colour module genes (Ensembl ID).

**Table S2:** Colour module gene enrichment analysis results.

**Table S3:** Lists of FPKM values corresponding to 33,599 genes.

**Table S4:** Lists of TPM values corresponding to 33,599 genes.

**Table S5:** Lists of DEGs (Symbol ID) selected based on adj *p* below 0.05.

**Table S6:** Lists of differential gene KEGG enrichment analysis results (*Q* values below 0.05).

**Table S7:** Lists of 10 clusters of 33,599 genes under the MFUZZ.

**Table S8:** Lists of DEGs (Ensembl ID) between groups.

**Table S9:** Lists of common genes between DEGs and 10 MFUZZ clusters.

**Figure S1:** Sequencing Data Quality Control Evaluation. Each of the 12 samples has a data size greater than 6G. All samples have Q20 above 98% (the lowest not below 90%) and Q30 above 94% (Q30 requirement is above 85%, the lowest should not be below 80%).

**Figure S2:** Classification of raw reads. The sequencing data filtration of PRO1 can be seen that out of the 20,777,010 raw reads, in percentage, 93.71% are clean reads and 5.21% reads related to the adapter sequence. The sequencing data filtration of PRO2 can be seen that out of the 22,937,280 raw reads, in percentage, 94.21% are clean reads and 5.08% reads related to the adapter sequence.

**Figure S3:** Analysis of network topology for various soft‐thresholding powers. The top panel shows the scale‐free fit index (*y*‐axis) as a function of the soft‐thresholding power (*x*‐axis). The down panel displays the mean connectivity (degree, *y*‐axis) as a function of the soft‐thresholding power (*x*‐axis). Soft Threshold: 19.

**Figure S4:** qRT‐PCR Validation of Differential Gene Expression in Mouse Endometrium Across Estrous Cycle Stages Panels show the relative mRNA expression levels of 12 selected differentially expressed genes (*Wnt5a*, *Wnt7b*, *Hif1α*, *Hnf1α*, *Cdk1*, *Krt8*, *Hoxa10*, *Fgf9*, *Nid1*, *Nog*, *Nrk* and *Pdgfd*) measured by quantitative real‐time PCR (qRT‐PCR). The expression data are presented as log_2_‐transformed relative expression values and are compared alongside corresponding RNA sequencing results to confirm consistency in gene expression trends across estrous cycle phases.

**Figure S5:** GO‐CC and GO‐CC multi‐group enrichment analysis results of inter‐group differential genes (DEGs).

## Data Availability

The data presented in the study were deposited in the BioProject under accession number PRJNA1378782 (https://www.ncbi.nlm.nih.gov/bioproject/?term=PRJNA1378782). The data that support the findings of this study are available from the corresponding author upon reasonable request.
