# Supplementary material for: Dynamic Transcriptomic Profiling of Mouse Endometrium Across the Estrous Cycle Reveals Phase‐Specific Regulatory Networks Underlying Cyclic Remodelling
Source: J Cell Mol Med. 2026 Jun 26;30(12):e71265. doi: 10.1111/jcmm.71265 (PMC13309392; doi:10.1111/jcmm.71265)
Supplement: Supplementary file 10 — Figure S1: Sequencing Data Quality Control Evaluation. Each of the 12 samples has a data size greater than 6G. All samples have Q20 above 98% (the lowest not below 90%) and Q30 above 94% (Q30 requirement is above 85%, the lowest should not be below 80%). [file JCMM-30-e71265-s005.pdf]

sample	library	raw_reads	clean_reads	clean_bases	error_rate	Q20	Q30	GC_pct
**PRO1**	FRAS202078901-1a	42948272	41554020	6.23G	0.02	98.31	**95.01**	48.47
**PRO2**	FRAS202078903-1a	47284426	45874560	6.88G	0.02	98.26	**94.96**	49.66
**PRO3**	FRAS202078905-1a	45276908	44259340	6.64G	0.02	98.02	**94.37**	49.95
**E1**	FRAS202078906-1a	47719618	46549132	6.98G	0.02	98.18	**94.47**	49.44
**E2**	FRAS202078908-1a	46101944	45072774	6.76G	0.02	98.27	**94.94**	49.72
**E3**	FRAS202078910-1a	46651662	45442650	6.82G	0.02	98.01	**94.35**	48.86
**MET1**	FRAS202078912-1a	46571794	45429284	6.81G	0.02	98.19	**94.77**	48.85
**MET2**	FRAS202078914-1a	44738838	43532522	6.53G	0.02	98.04	**94.35**	49.51
**MET3**	FRAS202078916-1a	44657328	43608798	6.54G	0.02	98.03	**94.37**	49.3
**DI1**	FRAS202078918-1a	46350594	44584842	6.69G	0.02	98.17	**94.79**	48.73
**DI2**	FRAS202078920-1a	45631676	44939158	6.74G	0.02	98.18	**94.62**	49.38
**DI3**	FRAS202078922-1a	47674754	46614902	6.99G	0.02	98.11	**94.55**	49.27
