# Supplementary material for: Dynamic Transcriptomic Profiling of Mouse Endometrium Across the Estrous Cycle Reveals Phase‐Specific Regulatory Networks Underlying Cyclic Remodelling
Source: J Cell Mol Med. 2026 Jun 26;30(12):e71265. doi: 10.1111/jcmm.71265 (PMC13309392; doi:10.1111/jcmm.71265)
Supplement: Supplementary file 11 — Figure S2: Classification of raw reads. The sequencing data filtration of PRO1 can be seen that out of the 20,777,010 raw reads, in percentage, 93.71% are clean reads and 5.21% reads related to the adapter sequence. The sequencing data filtration of PRO2 can be seen that out of the 22,937,280 raw reads, in percentage, 94.21% are clean reads and 5.08% reads related to the adapter sequence. [file JCMM-30-e71265-s002.pdf]

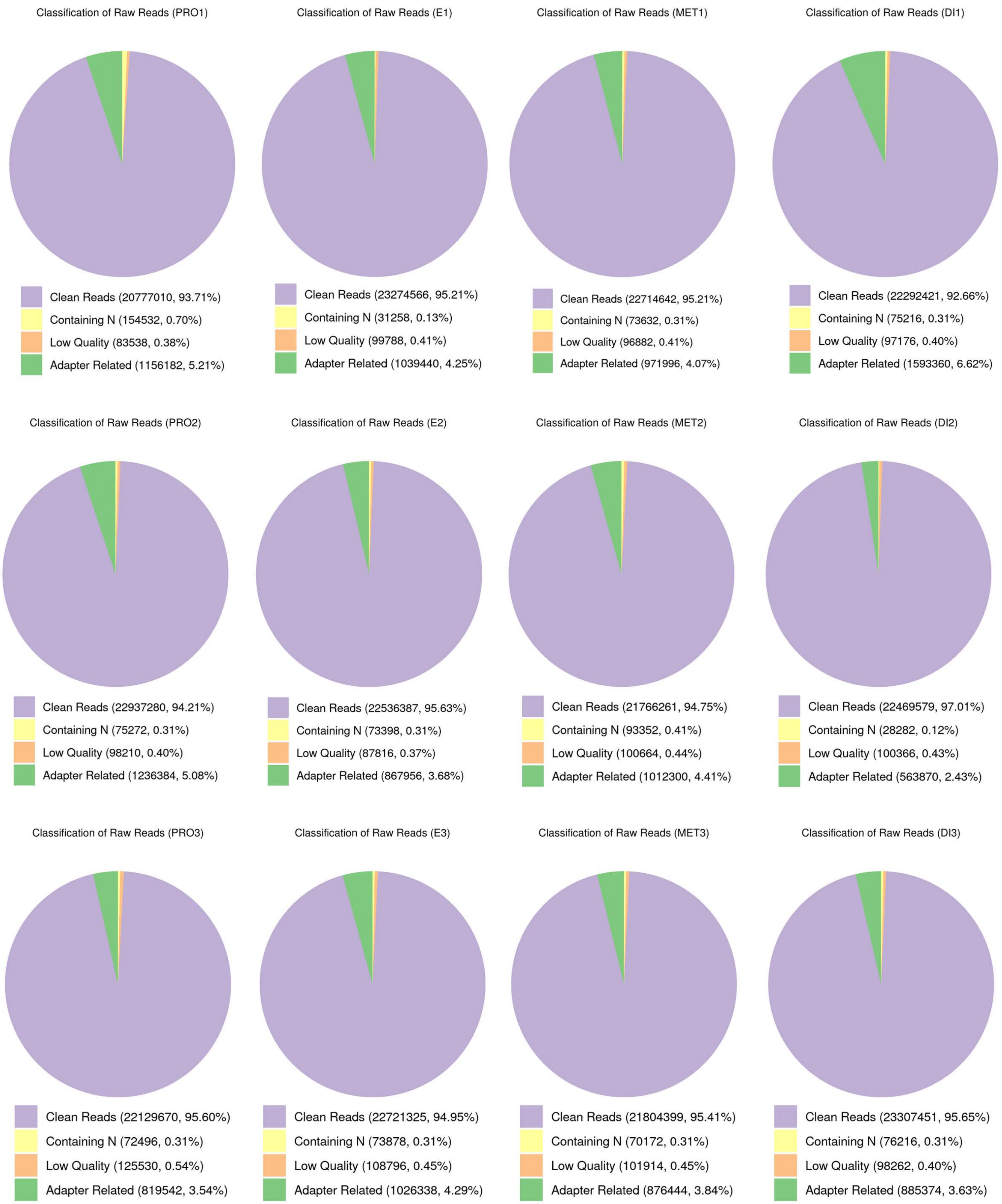

Classification of Raw Reads (PRO1)
Clean Reads (20777010, 93.71%)
Containing N (154532, 0.70%)
Low Quality (83538, 0.38%)
Adapter Related (1156182, 5.21%)
Classification of Raw Reads (E1)
Clean Reads (23274566, 95.21%)
Containing N (31258, 0.13%)
Low Quality (99788, 0.41%)
Adapter Related (1039440, 4.25%)
Classification of Raw Reads (MET1)
Clean Reads (22714642, 95.21%)
Containing N (73632, 0.31%)
Low Quality (96882, 0.41%)
Adapter Related (971996, 4.07%)
Classification of Raw Reads (DI1)
Clean Reads (22292421, 92.66%)
Containing N (75216, 0.31%)
Low Quality (97176, 0.40%)
Adapter Related (1593360, 6.62%)
Classification of Raw Reads (PRO2)
Clean Reads (22937280, 94.21%)
Containing N (75272, 0.31%)
Low Quality (98210, 0.40%)
Adapter Related (1236384, 5.08%)
Classification of Raw Reads (E2)
Clean Reads (22536387, 95.63%)
Containing N (73398, 0.31%)
Low Quality (87816, 0.37%)
Adapter Related (867956, 3.68%)
Classification of Raw Reads (MET2)
Clean Reads (21766261, 94.75%)
Containing N (93352, 0.41%)
Low Quality (100664, 0.44%)
Adapter Related (1012300, 4.41%)
Classification of Raw Reads (DI2)
Clean Reads (22469579, 97.01%)
Containing N (28282, 0.12%)
Low Quality (100366, 0.43%)
Adapter Related (563870, 2.43%)
Classification of Raw Reads (PRO3)
Clean Reads (22129670, 95.60%)
Containing N (72496, 0.31%)
Low Quality (125530, 0.54%)
Adapter Related (819542, 3.54%)
Classification of Raw Reads (E3)
Clean Reads (22721325, 94.95%)
Containing N (73878, 0.31%)
Low Quality (108796, 0.45%)
Adapter Related (1026338, 4.29%)
Classification of Raw Reads (MET3)
Clean Reads (21804399, 95.41%)
Containing N (70172, 0.31%)
Low Quality (101914, 0.45%)
Adapter Related (876444, 3.84%)
Classification of Raw Reads (DI3)
Clean Reads (23307451, 95.65%)
Containing N (76216, 0.31%)
Low Quality (98262, 0.40%)
Adapter Related (885374, 3.63%)
