# Supplementary figures and images for: Dynamic Transcriptomic Profiling of Mouse Endometrium Across the Estrous Cycle Reveals Phase‐Specific Regulatory Networks Underlying Cyclic Remodelling
Source: J Cell Mol Med. 2026 Jun 26;30(12):e71265. doi: 10.1111/jcmm.71265 (PMC13309392; doi:10.1111/jcmm.71265)

Scale independence

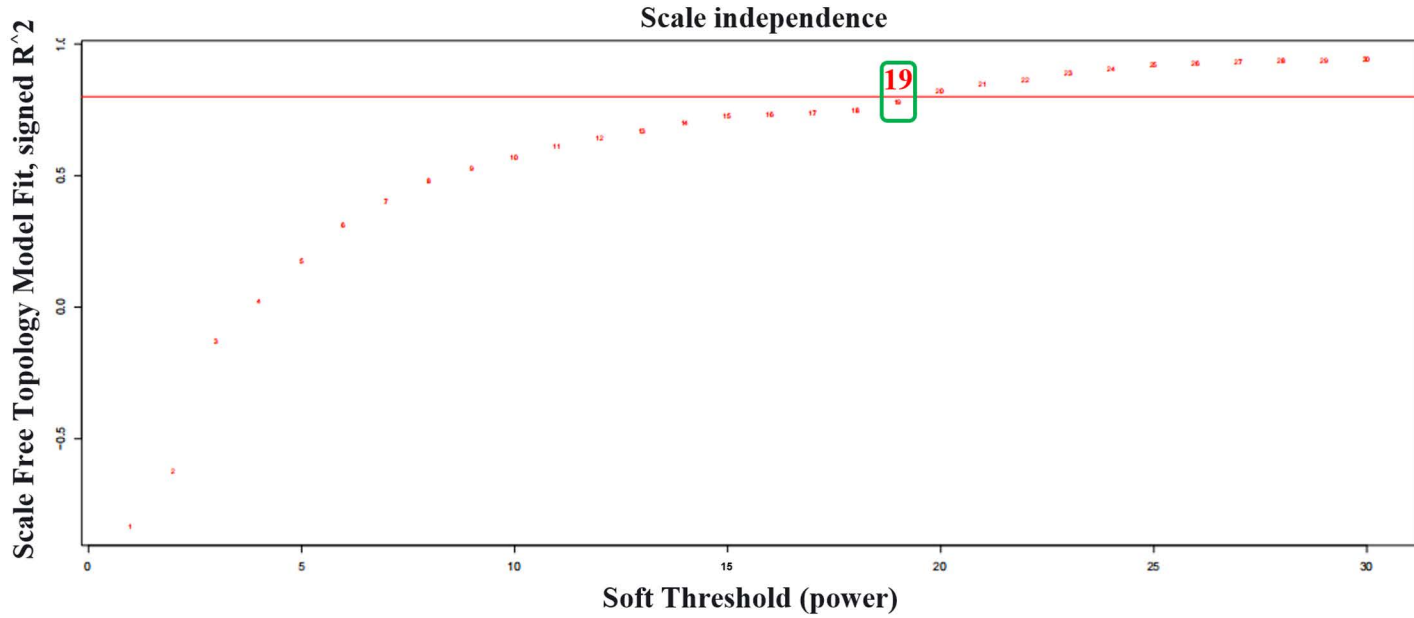

Mean Connectivity

Soft Threshold (power)

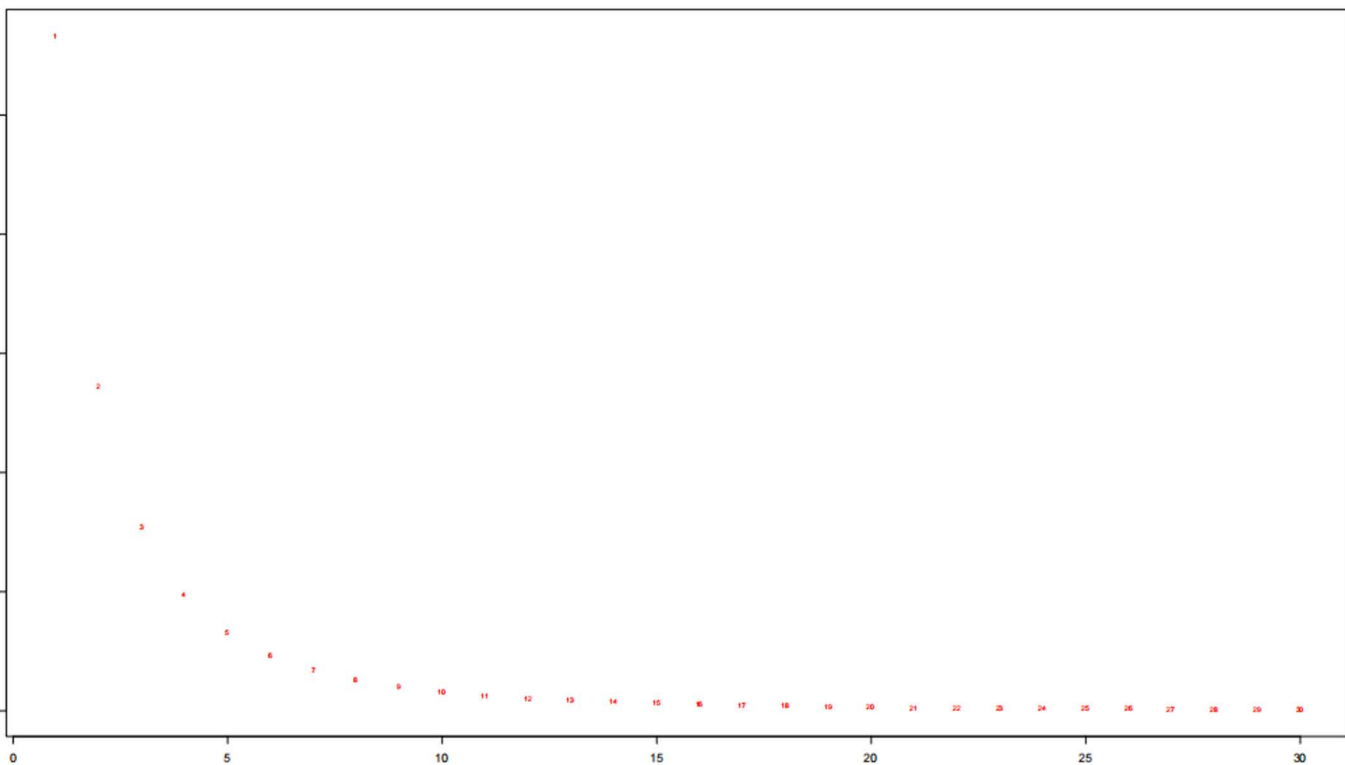

Supplement: Supplementary file 12 — Figure S3: Analysis of network topology for various soft‐thresholding powers. The top panel shows the scale‐free fit index (y‐axis) as a function of the soft‐thresholding power (x‐axis). The down panel displays the mean connectivity (degree, y‐axis) as a function of the soft‐thresholding power (x‐axis). Soft Threshold: 19. [file JCMM-30-e71265-s006.pdf]

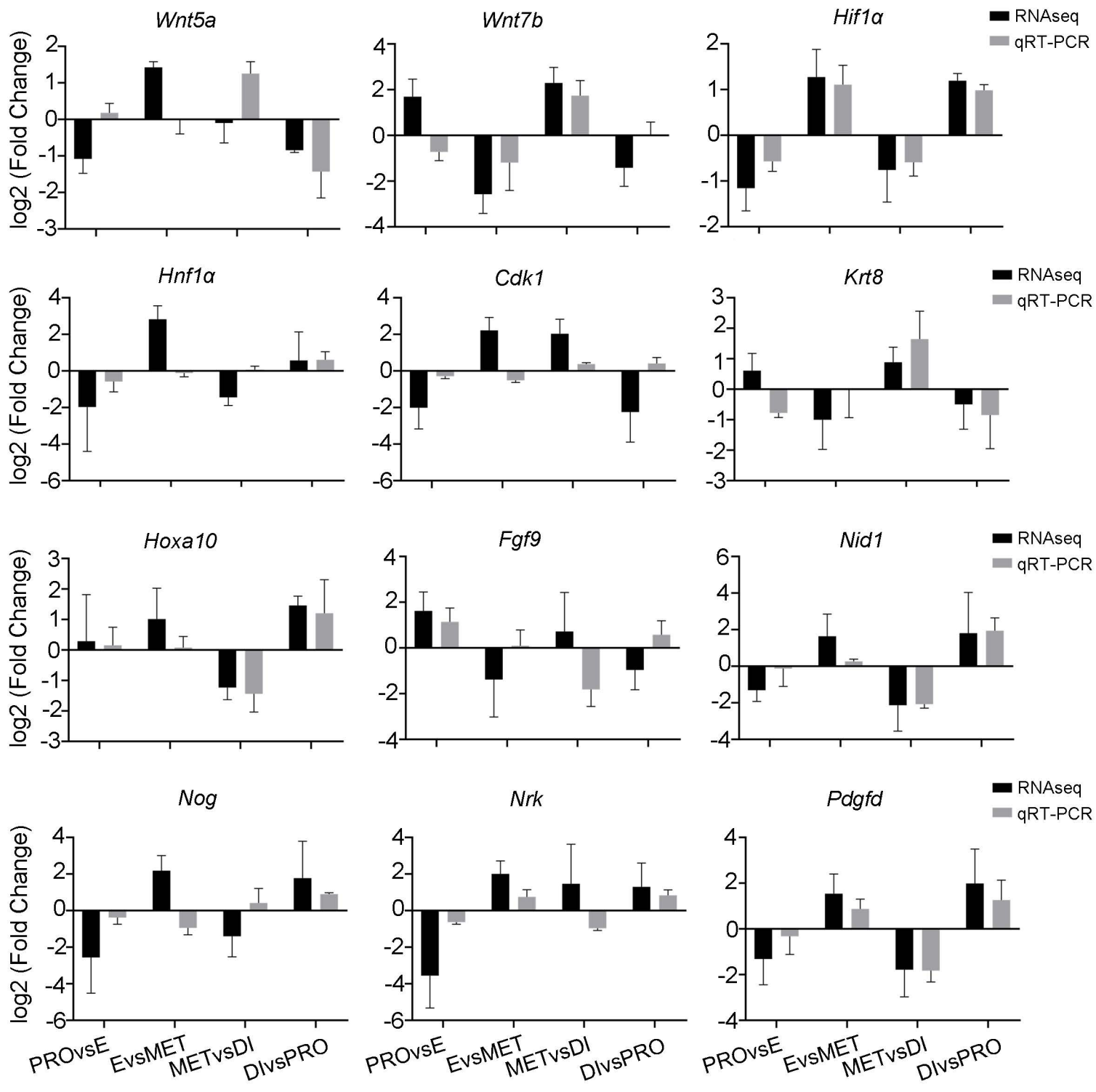

Supplement: Supplementary file 13 — Figure S4: qRT‐PCR Validation of Differential Gene Expression in Mouse Endometrium Across Estrous Cycle Stages Panels show the relative mRNA expression levels of 12 selected differentially expressed genes (Wnt5a, Wnt7b, Hif1α, Hnf1α, Cdk1, Krt8, Hoxa10, Fgf9, Nid1, Nog, Nrk and Pdgfd) measured by quantitative real‐time PCR (qRT‐PCR). The expression data are presented as log2‐transformed relative expression values and are compared alongside corresponding RNA sequencing results to confirm consistency in gene expression trends across estrous cycle phases. [file JCMM-30-e71265-s004.pdf]

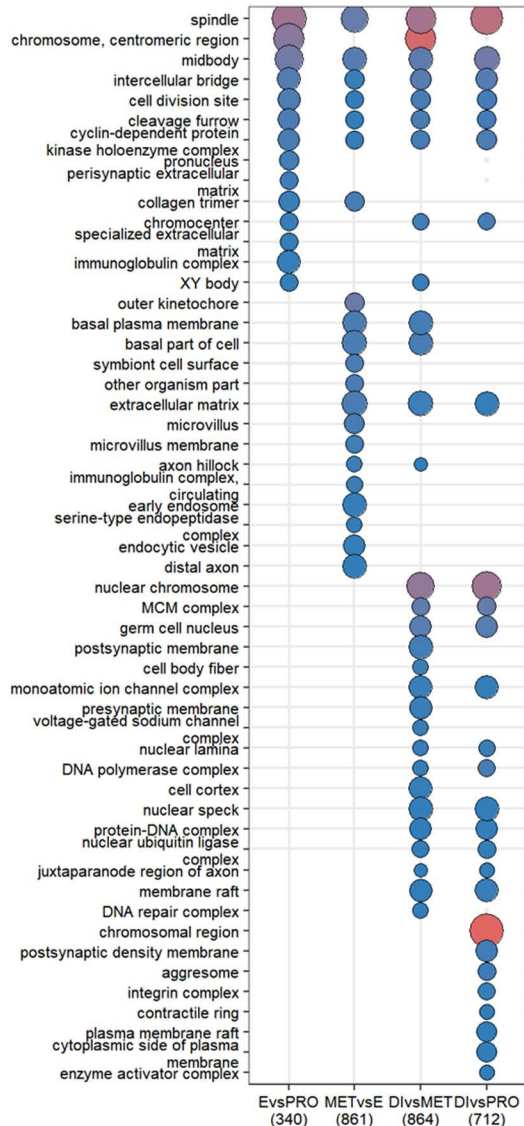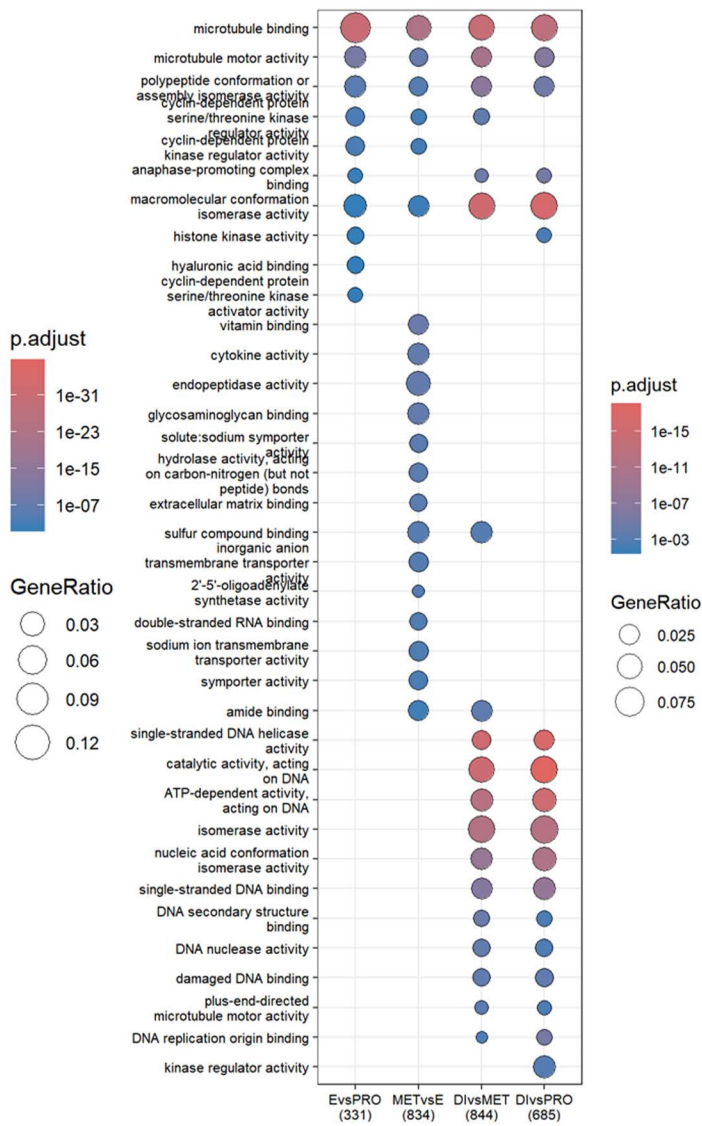

Supplement: Supplementary file 14 — Figure S5: GO‐CC and GO‐CC multi‐group enrichment analysis results of inter‐group differential genes (DEGs). [file JCMM-30-e71265-s011.pdf]
